# Overcoming Male Factor Infertility: A Journey Through Assisted Reproductive Technology With Platelet-Rich Plasma Therapy

**DOI:** 10.7759/cureus.55378

**Published:** 2024-03-02

**Authors:** Shradha M Ulhe, Namrata Choudhary, Jarul Shrivastava, Shilpa Dutta, Sudhanshu M Dakre, Akash More

**Affiliations:** 1 Clinical Embryology, Datta Meghe Institute of Higher Education and Research, Wardha, IND

**Keywords:** infertility, prp, opu, dna fragmentation, oocyte, sperm

## Abstract

This case study presents a couple’s journey through assisted reproductive technology (ART) experiencing two failed in vitro fertilization cycles. The couple underwent a comprehensive examination, revealing the normal parameters for the female, but asthenoteratozoospermia in the male indicating high morphological defects and reduced sperm motility. Subsequently, intracytoplasmic sperm injection (ICSI) was planned. Despite retrieving six oocytes during ovum pickup (OPU), all blastocysts stopped growth on the second day, prompting a sperm chromatin test disclosing highly DNA-fragmented sperm. Platelet-rich plasma (PRP) therapy was initiated to improve sperm quality, along with frozen embryo transfer (FET). Sperm were incubated with PRP, yielding improved sperm motility and reduced sperm DNA fragmentation. OPU yielded five good-quality metaphase II (MII) oocytes, which were successfully fertilized with PRP-treated sperm, resulting in the formation of four blastocysts. These blastocysts were frozen and later used for FET, resulting in a positive pregnancy outcome and successful conception. This case highlights the importance of personalized intervention in addressing the infertility factor in males and achieving successful ART outcomes.

## Introduction

According to the WHO (2023), "infertility is defined as a condition of the female or male reproductive system which is defined by the failure to achieve pregnancy after one year of unprotected sexual intercourse." In couples with infertility, around 85% have identifiable causes, while the cause remains unknown in the remaining 15% of couples [1]. Ovulatory dysfunction, tubal disease, and male factor infertility are some of the common causes of infertility. Modifications in lifestyle and environmental factors such as alcohol consumption, obesity, and smoking can negatively affect fertility [1]. According to the WHO, nine out of every 100 couples face fertility problems, with half of these cases involving issues related to male fertility. There are many reasons that cause infertility in males, ranging from medical illnesses or medications to lifestyle choices and genetic mutations [2]. Urogenital infections caused by viruses or bacteria, or occasionally by parasites, target the male accessory glands, seminal tract, and testicles, leading to increased oxidative stress and inflammation. These infections alter sperm parameters and increase sperm DNA fragmentation (SDF) [3].

SDF occurs when the DNA in sperm cells gets damaged; it is one of the main causes of male infertility. SDF is caused by reactive oxygen species (ROS), apoptosis, and failures in the protamination process [4]. A high DNA fragmentation index (DFI) (DFI ≥ 30%) reduces the rate of biochemical pregnancy, the clinical pregnancy rate of frozen embryo transfer (FET), and the delivery rate of fresh embryo transfer [5]. Autologous platelet-rich plasma (PRP) is used at a 2% concentration to decrease ROS, significantly improving sperm parameters, reducing ROS and DNA fragmentation, and improving motility [6]. PRP is often used to treat infertility in females but is rarely utilized for men with similar issues. However, studies suggest that the growth factors and hormones in PRP might improve sperm quality. It has been found that treating semen samples with PRP therapy significantly improves sperm quality, including morphology, progressive morphology, motility, concentration, and the percentage of sperm with good fertilization ability. This indicates that using PRP to incubate semen samples could be a useful treatment alternative for males with abnormal semen parameters undergoing procedures like intrauterine insemination (IUI) or in vitro fertilization (IVF) [7]. This case presents a male undergoing assisted reproductive technology (ART) treatment with PRP therapy.

## Case presentation

Patient information

A couple experiencing primary infertility for five years visited an ART clinic situated in the rural region of Wardha. The female was 36 years old, and the male partner was 45 years old. They had a history of two failed IVF cycles.

Medical history and physical examination

The patients had no prior surgical intervention and there was no recommendation for future surgical procedures. Both the male and female had normal body mass index (BMI) values, with the male having 25 kg/m² and the female having 23 kg/m².

Diagnostic assessment

The couple underwent certain hormonal tests and physical examinations to investigate the cause of infertility.

Male

Scrotal ultrasound was conducted as a part of the physical examination, but no abnormality was observed in the result. Hormonal tests, including luteinizing hormone (LH) and follicle-stimulating hormone (FSH), were found to be within the normal range as represented in Table 1.

**Table 1:** Hormonal profile of the male partner. LH: luteinizing hormone; FSH: follicle-stimulating hormone; mIU/L: milli-international units per liter.

Hormones	Observed level	Reference level
LH	2.4 mIU/mL	1.7-8.6 mIU/mL
FSH	3.7 mIU/mL	1.5-12.4 mIU/mL

However, semen analysis revealed a low count of progressively motile and morphologically normal sperm, indicating the problem of asthenoteratozoospermia, as shown in Table 2.

**Table 2:** Semen parameters of the male. ml: milliliter; mil/ml: million per milliliter; pH: potential of hydrogen.

Parameter	Observed limit	Reference limit
Semen volume	1.5 ml	1.5-2.0 ml
Morphological defects	97%	96% or less
Normal morphology	3%	4-14%
Vitality	46%	58% or more
Progressive motility	27%	32% or more
Count	20 mil/ml	15 million/ml or more
pH	7.3	7.2-7.4
Color	Opaque white	Opaque white
Viscosity	Liquefied within 20 minutes	Should liquefy ≤ 20 minutes

Female

Hormonal tests, including FSH, anti-Müllerian hormone (AMH), LH, and thyroid-stimulating hormone (TSH), were conducted, and all the hormonal levels were found to be in the normal range, as displayed in Table 3.

**Table 3:** Hormonal profile of the female partner. TSH: thyroid-stimulating hormone; AMH: anti-Müllerian hormone; FSH: follicle-stimulating hormone; ng/ml: nanograms per milliliter; mIU/L: milli-international units per liter; IU/L: international units per liter.

Hormones	Observed level	Reference levels
Progesterone	11 ng/ml	10 ng/ml and more
TSH	1.3 mIU/L	0.4-2.34 mIU/L
AMH	2.2 ng/ml	1-4 ng/ml
FSH	5.3 IU/L	4.7-21.5 IU/L

Additionally, ultrasound sonography was performed to check for any structural abnormalities, but no abnormality was observed. This result suggests that the female does not have any infertility-causing factors.

Treatment

Both the male and female underwent the required examination to identify the cause of infertility. The female’s assessments revealed normal parameters, while the male was diagnosed with asthenoteratozoospermia, indicating sperm with reduced motility and high morphological defects. As a result, conventional intracytoplasmic sperm injection (ICSI) was planned and the ovum pickup (OPU) was scheduled accordingly. Before OPU, the female underwent progesterone stimulation for a specific duration.

**Figure 1:**
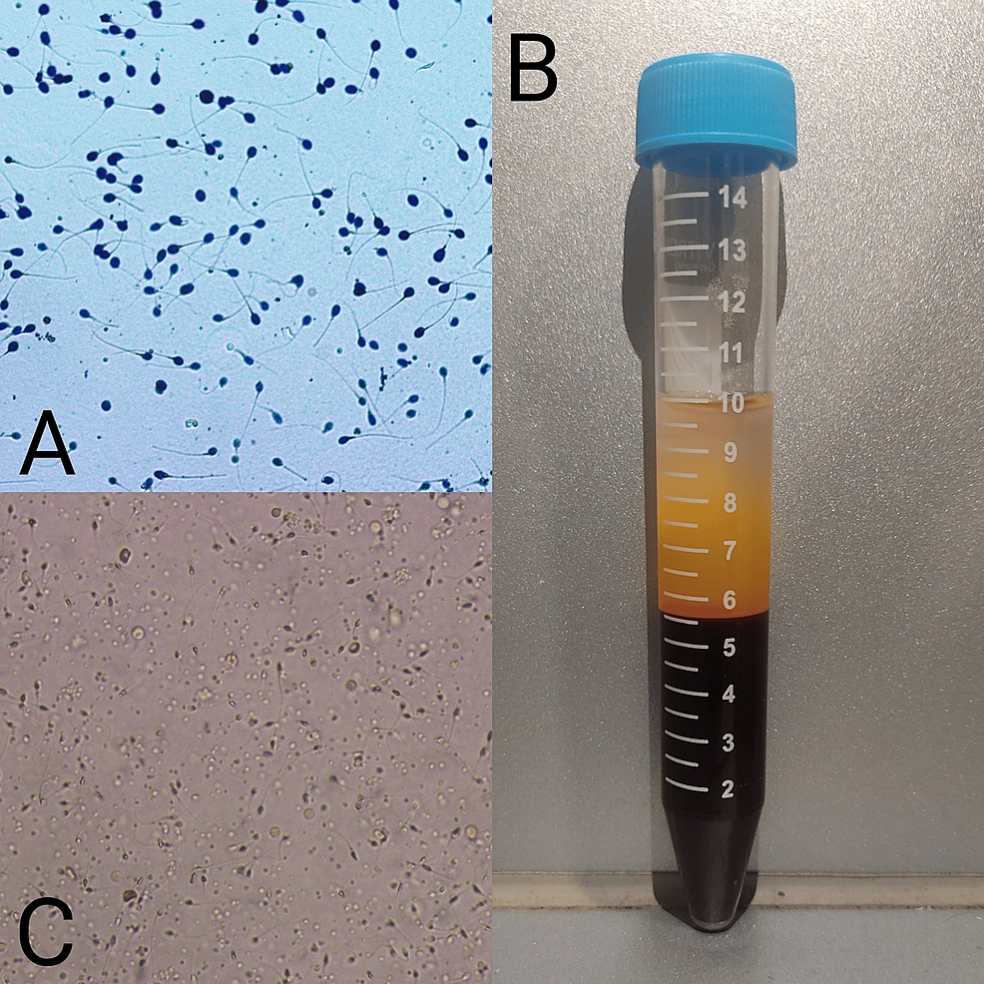
DNA-fragmented sperm and sperm incubated with PRP. A: DNA-fragmented sperm. B: Plasma separated from the blood (the upper yellowish layer is the plasma while the lower dark red layer is red blood cells. C: Sperm incubated in platelet-rich plasma (PRP).

OPU was performed as scheduled, during which six oocytes were retrieved, four of which were of good quality. These oocytes were then injected with sperm and cultured, but on the second day, the growth of all blastocysts was observed to be arrested. This suggested a potential issue with either the oocytes or the sperm. To point out the actual cause, a sperm chromatin test was performed to check the SDF. The test revealed highly DNA-fragmented sperm as presented in Figure 1, which resulted in the arrest of blasts development.

**Figure 2:**
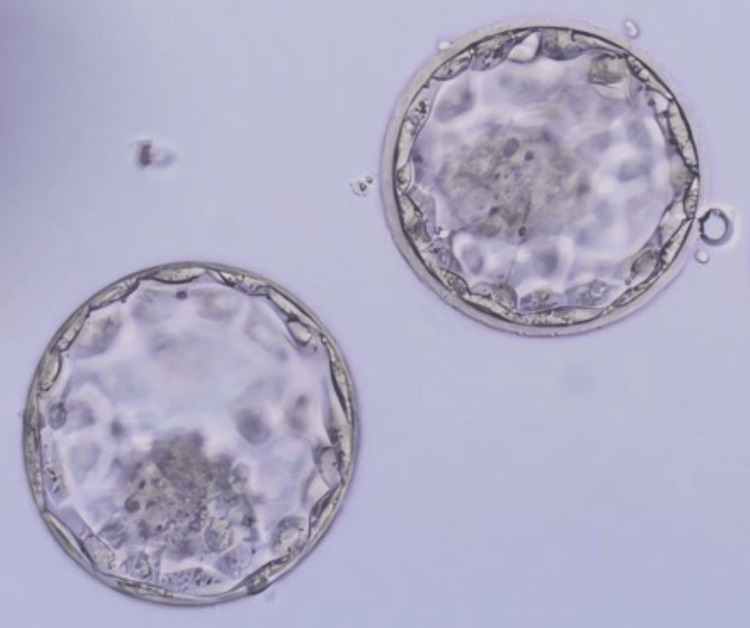
Day five blastocyst stage embryo transferred to the patient on day six of progesterone.

Consequently, a decision was made to pursue PRP therapy as a potential intervention intended to enhance sperm quality and perform FET. Firstly, the semen sample was collected from the male and processed by performing a simple wash method, in which the sperm were centrifuged and separated from other seminal components. Then, around 10 ml of blood was collected from the male using a 10 ml syringe, and the platelets were separated from the blood by spinning it into the centrifuge. The sperm were then overlaid with a 2% concentration of PRP and then incubated for an hour as displayed in Figure 1. After one hour, the sperm were examined, and significant results were observed in sperm, with improved motility and decreased sperm DNA fragmentation. Alongside the female partner was stimulated for OPU, and the OPU was performed, resulting in the retrieval of five good-quality metaphase II (MII) oocytes. These MII-quality oocytes were then injected with PRP-processed sperm, and cultured till day five, which resulted in four 4BA-quality blastocysts. These blastocysts were then frozen on two different cryofreezing devices (2+2) and after one month, FET was performed, transferring two blastocysts as shown in Figure 2. After the procedure, the female patient was advised to rest for a few hours before being discharged.

Follow up

After 14 days, the female partner was advised to check the β-human chorionic gonadotropin (β-hCG) level, which showed a positive sign for embryo implantation, indicating pregnancy with a level of 230 mIU/ml. Subsequently, the female was monitored to ensure adherence to given instructions and prescribed medicines throughout the complete pregnancy period.

## Discussion

This case demonstrates the complexity of addressing infertility issues through ART, especially in the cases of male factor infertility. Despite the female partner’s normal parameters, the diagnosis of asthenoteratozoospermia in the male highlighted significant difficulties. The decision to process with ICSI and subsequent OPU revealed challenges with blastocyst development, prompting further investigation into SDF. The identification of highly fragmented sperm led to the implementation of PRP therapy to improve sperm quality. The successful utilization of PRP-processed sperm in conjunction with FET resulted in the development of good-quality blastocysts. This case underlines the importance of detailed examination and personalized treatment strategies in overcoming infertility obstacles. Additionally, it highlights the potential of advanced approaches like PRP therapy in improving fertility outcomes, providing hope for couples navigating the challenges of ART.

A study by Martinez et al. found that high SDF in males leads to lower blastocyst formation rates in IVF. Couples with a male partner aged 40 years or more also had a higher miscarriage rate [8]. In a study by Alvarez Sedo et al., it was revealed that a higher level of SDF can adversely affect blastocyst growth and pregnancy rate, even when using good-quality oocytes. Higher DNA fragmentation levels are associated with a higher possibility of embryonic growth arrest [9]. Similarly, in our case, blastocyst growth was arrested due to an elevated level of sperm DNA fragmentation (DFI: 40%).

In the study conducted by Nabavinia et al., PRP was found to effectively improve the progressive motility and viability of the sperm while decreasing the count of immotile sperm. Sperm viability was notably higher in the PRP-treated samples, and PRP significantly reduced the rate of abnormal sperm [10]. A study conducted by Yan et al. showed that adding 5% PRP to the frozen-thawed sperm improved sperm viability, motility, and membrane integrity. Also, there was a slight reduction observed in DNA fragmentation and the reactive oxygen species [11]. Similarly, when we incubated sperm with PRP, it resulted in a reduction of SDF and an increase in sperm motility.

## Conclusions

In conclusion, this case underscores the challenges faced by a couple undergoing ART due to male factor infertility. Despite failed IVF cycles, a tailored approach including ICSI and PRP therapy was employed, resulting in the successful development of viable blastocysts. To address sperm DNA fragmentation, the use of PRP therapy proved beneficial in improving sperm quality and ultimately contributing to the success of FET. This case highlights the importance of personalized treatment strategies in overcoming complex fertility issues.
